# Software-Defined Business: Implications for IT Management

**DOI:** 10.1007/s12599-020-00669-6

**Published:** 2020-10-27

**Authors:** Rainer Alt, Jan Marco Leimeister, Thomas Priemuth, Stephan Sachse, Nils Urbach, Nico Wunderlich

**Affiliations:** 1 Leipzig University, Leipzig, Germany; 2 University of St. Gallen, St. Gallen, Switzerland; 3 Universität Kassel, Kassel, Germany; 4 Head Production System, BMW Group Plant Leipzig, Leipzig, Germany; 5 Manager Digitalization, VNG AG, Leipzig, Germany; 6 Frankfurt University of Applied Sciences and Fraunhofer FIT, Frankfurt, Germany; 7 IT University of Copenhagen, Copenhagen, Denmark

## Introduction

Rainer Alt

In August 2011 the Wall Street Journal published an essay by Netscape co-founder Marc Andreessen titled “Why software is eating the world”. He argues that “more and more major businesses and industries are being run on software and delivered as online services—from movies to agriculture to national defense. Many of the winners are Silicon Valley-style entrepreneurial technology companies that are invading and overturning established industry structures. […] Companies in every industry need to assume that a software revolution is coming.” (Andreessen 2011). By pointing at examples in various industries from automotive and banking to defense, healthcare, music, retailing, and telecommunications, he illustrates the transformational power of information technology (IT) and software in particular. The discussion is not new and dates back to the early 2000s. For example, Power and Jerjian (2001, p. 99) mention for Heathrow airport that “software is the thing that runs the airport” and in a biological analogy they consider software to be the nervous system. Meanwhile, the large “big tech” software companies (e.g., GAFAM, BATX)^1^ have impressively confirmed this development: their digital platforms have spread over many industries with an impact on processes (e.g., interaction and transaction), on products (e.g., app stores and services) as well as on business models (e.g., streaming and as-a-service models). The recent tech-portmanteaus (e.g., Fintech, Insurtech, Regtech) also reflect the attitude of these startup businesses, which perceive themselves rather as IT (tech) companies than as representatives of the industry they are aiming to transform (Meijer and Kapoor 2014). In the same vein, the high valuation of Tesla Motors since early 2020 spurred an intense debate of whether the company is in the automotive or the tech business (e.g., Klebnikov 2020).

Obviously, these developments have numerous implications for the field of IT management, which were discussed during a panel at the 82nd Annual German Business Researcher Conference on March 18, 2020. The panel builds on prior discussions regarding the impact of digitalization on IT management. For example, a BISE discussion section in 2017 addressed the implications of the broad trend of digitalization for the field of Business Information Systems Engineering and pointed out new topics, such as digital innovation and transformation, which complement existing competencies (e.g., in modeling and managing enterprise IT). The discussion revealed strong arguments for the strategic role of IT and that business and IT departments as well as academia and practice need to collaborate more closely (Legner et al. 2017). While these statements adopted a rather broad perspective, another discussion section focused on “the impact of digitalization on the IT department” (Urbach et al. 2019). It mentioned important and intricate challenges: On the one hand, IT departments need to move closer to the business units and, on the other hand, they need to constantly update their technological skills in an environment with a high pace of technological innovation. In a world “eaten by software” these developments are equally prevalent and lead to the question how businesses address these challenges, in particular, how they assess the dominant role of software, how activities in managing the software lifecycle should be organized, and what the implications are for the BISE community.

### Ubiquity of Software

To set the stage, some basics regarding software shall be introduced. Together with the hardware, software constitutes a computer system and comprises “computer programs, procedures and possibly associated documentation and data pertaining to the operation of a computer system.” (IEEE 1990, p. 66). In today’s world of miniaturized and networked hardware (“Internet of Things”), most physical objects have become computer systems and are coordinated by software. However, researchers observe a reversed relationship between hardware and software: increasingly, it is the software that defines the hardware and that leads to differentiation in the market (Zhu et al. 2016). While the complexity of software is growing, hardware in many areas tends to be more standardized and simplified. For example, Tesla is aiming to reduce the complexity of a car’s body from now 70 parts to four in the forthcoming Model Y and eventually plans only one part (Hampel 2020). In contrast, each Tesla software update comprises additional functionalities, recently an assistant that recognizes traffic lights and stop signs. Enhancing physical objects with software and making them amenable to being coordinated virtually is only a first implication. It leads to a second implication, which allows physical objects to be complemented with services, thus resulting in smart service systems (e.g., Beverungen et al. 2019), which themselves consist of software. Examples of this servitization are manifold, for example, many car manufacturers now provide a variety of services from emergency assistance to car sharing. A third direction marks the substitution of physical objects with software, which has taken place early on, for instance the replacement of answering machines by voicemail service or that of physical music records by MP3 files. Thus, these developments support the vigor of software-defined business where material and immaterial resources may be managed electronically regardless of their location. Due to this ubiquity, Zhu et al. (2016) even coined the term software-defined anything, which is used in a more focused way in the notion of software-defined businesses. It extends the technological concepts of software-defined data center, software-defined network and software-defined storage, which strive for on-demand availability and efficient (re)configuration, in the business domain (Strikeleather 2017, p. 84 f). In this sense, a software-defined business is an application-oriented interpretation and denotes organizations whose value creation (via processes, products, business models) strongly depends on software and services.

### IT Management in Software-Defined Businesses

If software affects business processes, products, and business models alike, the question is how software-defined businesses can be organized to meet these developments. Traditionally, the obvious responsibility for these topics will be with IT management, which is recognized as an enterprise function that is concerned with planning, organizing, and controlling IT resources such as people, processes, technology, and data (e.g., Keel et al. 2007; Cragg et al. 2013). Software, applications or IT services often range as one important area within the IT management or the CIO department, where the responsibilities for development and operations as well as for managing contacts with third-party software providers and the provision of the necessary development tools and environments are located. Although approaches in IT service management (e.g., ITIL, COBIT) have transformed IT departments into internal service providers that support line departments with (innovative) IT skills, the collaboration between the business and the IT side has remained difficult and led to statements like “IT is an island” (Peppard 2007) or the “business-IT divide” (Rahimi et al. 2016). Therefore, the question “Whose responsibility is IT management?” (Boynton et al. 1992) also applies to the software responsibilities and skills. In general, they might be centralized as well as decentralized within or without the organization’s IT management function. Among current initiatives are:

- *Decentralization* Along the lines of digitalization, new approaches may be required that recognize digital competences as an inherent element in all organizational units. This direction is fostered by software development environments that only require basic configuration skills such as no-code and low-code solutions. It may be assumed that this empowers members in line organizations to already automate simple routine activities, but apparently it also brings along the challenges of shadow IT (e.g., Klotz et al. 2020).
- *Centralization* Another path is to bundle software responsibilities in a separate department. For example, Volkswagen AG has created the new car.software organization unit in 2020 after having added a new function to its corporate board with the responsibility for group software activities (Volkswagen 2019). By “spinning off” the software responsibility from IT management into a dedicated organizational unit, software has arrived at eye level with the carmaker’s traditional functional departments such as research and development, production and logistics, or sales and marketing.
- *Agility* Methodologies in software engineering have evolved from the waterfall to more iterative approaches, which also involve the user, i.e. the business side. For example, Scrum as a popular agile framework has explicitly defined the role of the product owner as a part of Scrum teams, which jointly deliver small results at short intervals. Recent developments, such as DevOps, enhance this thinking of continuous deliveries in the direction of operations as well as innovation (e.g., Alt et al. 2019). In combination with a (digital) delivery pipeline, these are important prerequisites within companies to meet the customer expectations of continuous innovation, although the speed between the IT and line departments will often differ.
- *Sourcing* In view of constantly emerging new technologies, software development calls for a permanent mastery of new hardware and software. Specialized software companies and as-a-service providers should be recognized as partners in the software value chain (e.g., Ågerfalk et al. 2015). Similar to other functional competencies (e.g., manufacturing), there is a challenge of defining the own core competencies and striking partnerships with external specialists from the software industry. Finding a suitable degree of insourcing and outsourcing will be important in the field of software as well.

These directions are not mutually exclusive and will require each organization to define their individual configuration. To collect various views on the current state as well as on future developments, the panel brought together representatives from academia and practice. They agree on the role of software in general, but emphasize different aspects on the relationship between IT and line departments, the relationship of analog and digital businesses, the integration of internal and external resources as well as the on the role of research and education.

## Software Eats the World: The Role of Digital Value Creation for BISE Practice and Research

Jan Marco Leimeister

“Software eats the world” as a slogan from the early 2000s has often been attributed to Marc Andreessen, one of the most successful investors in the Silicon Valley venture scene. It has many facets: From a technological perspective, we have been seeing steep technological advances and innovations in hardware, algorithms, computing power, and data storage that have led to the rise of the cloud, the Internet of Things and also Big Data and Artificial Intelligence during the last two decades. The more our software eats the world, the more we enable the logic of software-based value creation to spread and evolve. From a value creation perspective, softwarization, understood as the use of a software solution, rather than traditional hardware, to solve a problem, is so much more than just the automation of existing processes and business models. We see more and more digital value creation (e.g., leveraging on marginal cost of zero, network effects, lock-in effects, etc.) to achieve exponential growth logic coming to areas that used to have traditional, linear value creation logics (growth is a function of input and marginal cost can never be below variable costs, etc.) (Leimeister 2020).

The advancement of digital environments to all our areas of business and private life enables entirely new designs and ways of value creation and value capturing, enabling much more attractive offerings to users and customers. Software and data are drivers for:

- “User-centricity”, enabling interactive value creation with each user at large scale, individualizing performance, price, etc. at any moment, anywhere, in any way (Brenner et al. 2014).
- “Everything as a service”, by modularizing all parts of a value network into partial services that can be offered (and combined) independently (Vargo et al. 2020).
- “Digital First”, by designing and controlling scalable and highly profitable digital core components (Bharadwaj et al. 2013), that allow the control of large value networks or ecosystems (such as, e.g., access to users, suppliers, etc.), frequently leading also to discontinuous innovations.
- “Always innovating”, by constantly and frequently testing all services with real users and in real markets using prototyping or minimum viable products or A/B tests, leading to unprecedented speed and scope of service innovations (Kohavi and Longbotham 2016).

These trends and ways of thinking about how to innovate constantly and differently, in the same way as most digital companies have innovated, often lead to superior offerings and entirely new business logics, therefore challenge almost all incumbents. How are incumbent organizations and their business and IT functions responding to this? And which research communities are tackling this in an adequate way? We can see at least two extreme scenarios:

- *IT eats Business* As companies digitize their business to greater extents, the more similarities they share with software or internet companies. Let us look at the example of a large private Swiss bank that, after several attempts to master “digital banking”, has recently decided to start an entirely new “Direct Banking” business unit to compete with the new players in the Fintech world. This digital business unit is headed by the former CIO of the bank, and it is run by a couple of hundred IT people and hardly any other staff—almost a revolution for the banking business in Switzerland. If the services of this direct banking unit are successful, they may easily be scaled up to be applied to all customers of the bank, across almost all markets the bank serves—with hardly more headcount needed. Therefore, the digital bank is run and managed by IT people, while applying the software business and digital value creation logic to the entire banking business.
- *Business eats IT* Volatile, uncertain, complex, and ambiguous (VUCA) environments increasingly characterize the current status of companies—and this is often considered to be the logical consequence of a more and more digital, interconnected, globalized world (Clegg et al. 2019). Improving organizational agility is one of the most frequently used approaches when trying to deal with such a “new normal”, a VUCA world. Such agile transformations are currently high on the agenda of many boards. Organizational agility is frequently translated as the ability to better sense what is happening on the markets, to customers and competitors, etc., and to better respond to changes as fast and adequately as possible (Tallon et al. 2019). This is a challenge that the software business has been facing for quite a while and which has led to the rise of agile work concepts such as Scrum. So many organizations now embrace such new work approaches originating from software/internet companies and apply them to their own non-IT units. We see concepts like DevOps, large scale Scrum frameworks (LESS, SAFE), etc., expanding from IT functions into the overall organization, trying to empower many of the elements of digital value creation for the overall organization. Thus, software eating the world leads to VUCA and digital value creation and innovation everywhere, and challenges organizational structures, processes, and business models alike.

Let us look at the example of a large Swiss-French insurance company. The agile transformation was the most critical strategic objective of the whole board. In order to empower the business to transform to agile work practices and new digital work modes, the CIO decided to place over 70% of his staff and more than 80% of the budget directly into the business units. This bold move is remarkable, as most managers do not easily give up parts of their area of responsibility. It means a key change to the existing structure of the company, breaking up silos of IT and business—we want to be all digital now, is the slogan, and this is another way to leverage the potential of digital value creation for an insurance company. The CIO is now also the chief business innovation officer, responsible for the complete new digital business.

In conclusion, many associated with the BISE research community are concerned about the current state of the discipline. While some researchers seem to address these challenges our counterparts in practice experience, others seem to be reluctant or bypass these topics. It is of utmost importance for the field, but also for businesses and society to find answers to the questions of how to leverage the potentials of digital technologies and digital value creation in all areas of our economy and society. Let us hope that the debaters’ positions contribute to an inspiring discourse on the future of the research on digital value creation in our discipline.

## The Individual Degree of Digitalization

Thomas Priemuth

Marc Andreessen’s article “Why Software Is Eating the World” (Andreessen 2011) describes impressively and with many examples the technological and economic change that has significantly shaped the corporate world over the last two decades. The facts that illustrate this trend speak a clear language. In 2019, seven of the world’s ten most valuable companies by market capitalization were Internet or IT companies (Microsoft, Apple, Amazon, Alphabet Inc., Facebook, Alibaba Group, and Tencent (Wikipedia 2020)). The business models and value creation of these companies focus primarily on digital products or services such as software, digital services, platforms and ecosystems, data and streaming services or the digital mediation of products, services, and advertising.

### Physical Products are Not Obsolete

Several developments may be observed. On the one hand, completely new digital products and services have emerged due to new technological possibilities. On the other hand, former analog products have completely migrated disruptively to digital offers or IT increasingly supports the processes for the production of goods and services. Andreessen describes an extreme change in the economy towards mostly digital companies. These companies are IT companies in terms of structure, organization, applied methods and how value is created. However, can these developments be generalized, and will they affect every company?

In recent years, the change described by Andreessen has not only shaped economies worldwide but has also had a significant impact on the viewpoint of every individual in the population. New business models for private customers and companies, extreme growth rates, gigantic company takeovers, very high market capitalizations and high returns are naturally the focus of the stock markets, the media and thus also in people’s personal perception. A focused perception automatically goes hand in hand with these facts, which are strongly in the economic and media spotlight. The focus is on the new, the front-runners, the game changers and moon shots.

To answer the question of how the trends described above affect existing and new companies in general, it is helpful to take a look at the structure of economies. Statistics show that in 2019 the “information and communication” sector accounted for 4.6% of the Germany GDP (Statistisches Bundesamt 2020). Approximately 3.5 million and thus 99.6% of all enterprises in Germany are small and medium-sized enterprises (SMEs), which account for 58% of net value added in Germany (BVMW 2020). In 2019, 19% of all German companies employed their own IT specialists. This means that over 80% of German companies do not have their own IT staff or IT organizations (Statista 2020). How do Andreessen’s statements and these statistics fit together?

Our everyday and professional life is already highly digitalized today and this trend will certainly intensify in the coming years. However, physical products or non-digital services will continue to account for a considerable proportion of this in the future. For the foreseeable future, people will have to eat something solid and drink something liquid, people will desire to travel to a real place and we are (yet) unable to beam goods or people to another place like in the movie classic “Star Trek”. We need food, a real existing home, machines to produce products, vehicles to transport people and goods, medical help in emergencies, people to care for the needy and much more. It should be remembered, that a large part of the value added in our societies will remain physical (or analog) products and services in the future.

### Softwarization Depends on Products and Services

However, depending on the branch of industry or service, IT and digitalization significantly support the processes of value creation, enrich them with new services and digital products or create new business models. To what extent are new and existing companies affected by these trends? The relevance for companies may be divided into three clusters, depending on which products or services represent the core performance of a company:

- *Products and services are primarily digital* In this case, new or existing products or services can be realized or conceivable in a completely digitalized form under the forecast of future technological developments. In these companies, the strategy, orientation, control and the necessary core competencies will clearly correspond to an IT/software company in the near future. If the core product today is still analog, the company must undergo a significant change towards the structures, process models, methods and architectures of an IT company. These products and services have experienced enormous growth in the last two decades, as they serve a previously non-existent segment. With increasing technical opportunities, this will continue in the coming years. However, the proportion of completely digitalized scopes accepted by society will slow down or become saturated in the long term.
- *The added value of physical/non*-*digital products is significantly supported by IT or digital services* The intended purpose of the products and services is based on a physical/non-digital core. However, important core processes such as production, finishing, marketing or sales are strongly supported by IT or by the integration of new services and products. The core task here is to strategically and precisely identify those areas that offer potential for IT- or software-supported improved efficiency or new value-added services. These will also be structured very similarly to IT-oriented structures. Depending on their strategic relevance, they need to be integrated into existing corporate structures either as internal services or as external services. In the future, the digital part could very well make up a considerable part or even the majority of the business model or company earnings. With regard to the mentioned statistical data, it is clear that this is the area in which most changes may be anticipated in the future.
- *Products or services that will retain their right to exist completely analog in the future and cannot be significantly supported by digital processes or services* The existence of these products and services may be based on several factors: First, a digitalization of the product or digital support of the value-added processes is technologically not possible. Second, the product/service is very strongly influenced by physical properties of objects such as their haptics and physical use. Third, the integration of digital elements is explicitly and deliberately avoided. Due to the already existing penetration of IT in the value chains of products and services, it is to be expected that this part will decrease strongly.

**Fig. 1:**
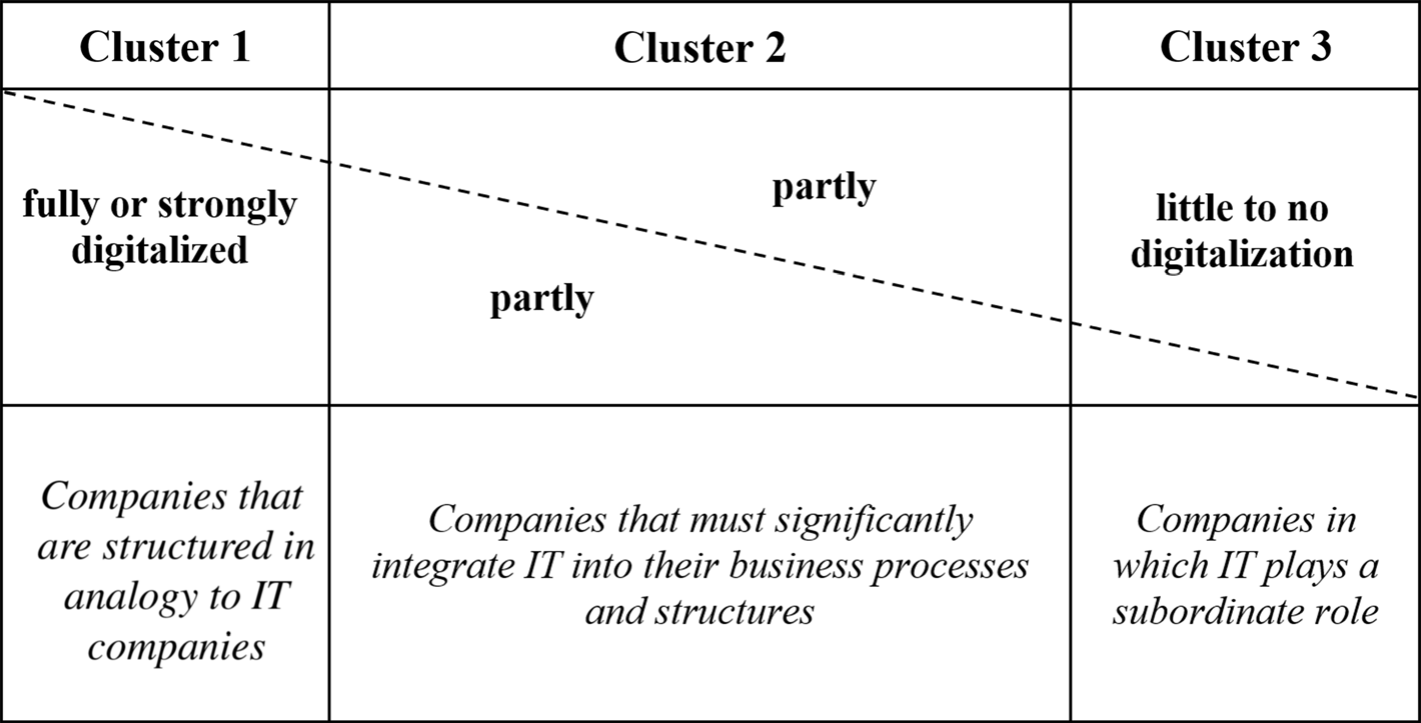
Potentials for softwarization in three clusters

A classification in these three clusters (see Fig. 1) is the challenge for new business ideas, but also for the strategic orientation of existing businesses, which every company has to answer correctly in order to continue to grow. Highly appropriate to this discussion is a contribution by Marc Andreessen, which he recently published on the website of his investment company. He criticizes the ability to manufacture physical products quickly and flexibly in the context of the Corona crisis. The article is aptly titled “It’s time to build” and he states that „We see this today with the things we urgently need but don’t have. We don’t have enough coronavirus tests, or test materials—including, amazingly, cotton swabs and common reagents. We don’t have enough ventilators, negative pressure rooms, and ICU beds […] Why do we not have these things? […] We could have these things but we chose not to—specifically we chose not to have the mechanisms, the factories, the systems to make these things. We chose not to *build*.“(Andreessen 2020).

This quote shows a different focus for Andreessen compared to his 2011 article, where the success of the companies described shows the power of change and the multitude of new options that IT offers. The descriptions from the current crisis also highlight the need to be able to produce analog and physical products efficiently and flexibly. A review from both perspectives should therefore be considered for future strategic orientations. A society needs to strike the right balance between being highly innovative and digitalized on the one hand and producing all the basic (and possibly non-digital) products it requires on the other.

### Conclusion

What relevance can be deduced from these considerations for the teaching of economic sciences? Future industrial engineers, business economists and managers do not need to be programming specialists. However, they do need a basic understanding of how IT works and of its potential, as well as basic IT architecture knowledge and the ability to understand and combine both worlds. The goal must be to enable students to transform this knowledge into strategy and then put it together to create a successful business model for the future of companies. It is therefore essential to convey a basic understanding of IT in the economic sciences. However, this should not only be deepened in academic education. The foundations for this should be laid during school education and anchored in the curriculum at an early stage.

In summary, there is no doubt that digitalization and IT are becoming increasingly important. They enjoy constant innovation and will continue to shape companies in terms of organization, structure and methods. At the same time, both analog and physical products and services will continue to retain their legitimacy. When looking at the three clusters above, it may be assumed that the second cluster will experience the greatest change. Depending on the influence of IT on the end product or its value chain, the degree of digitalization in companies varies greatly and must be assessed on a case-by-case basis.

## Empower the People–Empower the Company

Stephan Sachse

The ongoing discussion about technological trends, changing paradigms and the like do not have any significant impact on the economy if those concepts are not adopted by businesses. Bringing those ideas to life, in a valuable way, is subject to IT-management. The success of any efforts towards software-defined business is based on some key factors, which I believe are usually overlooked:

First, in a business context, IT is too important to leave it solely to the IT department. It needs a holistic approach and I have observed great accomplishments if the role of the CIO is joined by executives that are also responsible for business, financial, or HR departments. This allows a more aligned view on the plethora of subjects that are nowadays referred to as digital transformation. A transformation of a company, for instance towards a software-defined business, is a holistic change process for any incumbent company that has non-digital roots. Backed by C-level attention, a dedicated change management organization must be implemented. Every company that devises the challenges of that change process might conclude that this organization may better be established outside the IT department. From my experience, the non-technological aspects are the most difficult ones in that transformation process and are the ones that need the most persistence.

Second, the immanent transformation of business towards software-defined or at least digital-augmented companies is backed by current technological advances. However, it turns out that not the omnipresent trends such as blockchain or AI lead to a fundamental change in incumbent companies. There are more subtle trends that bring things forward: Becoming a more digital company requires a self-reflecting internal view. It is the inner “machine room” that needs to undergo a brush-up. Since value creation in most companies is driven by employees, the key is to advance the workforce. Besides streamlining and enhancing communication and collaboration infrastructures, I believe in the power of low-code and no-code-tools to augment a substantial amount of employees with next-level abilities. Enabling those in the company that run the processes and profoundly understand the business to solve everyday problems themselves, brings unknown benefits to the company: activities are accelerated, knowledge is accumulated, problems are no longer delegated, employees feel new meaning in their work—in short, a transformation process is initiated that shows immediate paybacks. I am still amazed by the 50+-year-old secretary that handled a plethora of everyday processes by hand until she was briefly introduced to MS PowerAutomate. Shortly after, she automated many ill-defined business processes on her own.

An implication of this trend is the decentralization of IT. Business units receive the tools to help themselves in many situations which are often not captured,e.g., by process charts. This is no new trend at all—just think of Excel scripts that have existed for decades and are utilized by “experts” in many business units. Low-code and no-code tools, in conjunction with cloud-based infrastructures, mark the next leap in the IT-abilities of business units. It comes with some new challenges—but I believe these challenges are worth their effort for any company.

Management processes must adapt to this new situation. The IT department is no longer the single point of solution for every process that needs to be automated. Moreover, this change unleashes resources for IT to cover more complex or more meaningful requirements in the future. For managers the main issue is to find their new role in appropriately governing that environment. They have to embrace a world where digital solutions emerge at any time within the company. Now they have to actively monitor and trace digital workflows and apps. No longer is a requirement brought to their attention before any action takes place. A company no longer runs the risk of being paralyzed by IT managers that seek their fortune in convenient management processes.

Third, the key to the sustainable transformation of businesses lies in the empowerment of the workforce. Any single worker must understand the necessity for new ways of work, lean and agile structures, and customer-centricity. Besides the provisioning of modern tools, like the above-mentioned low-code platforms, education and human resources development, is essential to create a truly digital company, outside of any showroom or innovation lab. Teaching a digital skillset first requires to be aware of the necessary skills and second to teach them at scale within the company.

Bringing these skills to life, and more importantly, keeping them alive is subject to the working culture. Progress must be embraced, mistakes must be allowed, initiative must be rewarded. Technology and tools that are introduced into a company without these prerequisites, skills, and this culture are condemned to fail. To conclude, becoming a software-defined business is all about toolset, skillset, and mindset. Each of these factors must be present throughout the entire company to the right degree. IT must be tightly woven into the business units and the classical IT department must evolve beyond the solution of humble everyday tasks that business units better deal with on their own. Whatever empowers the employee should eagerly be examined.

## The Digital Company: Moving from Software-Based to Software-Driven Management

Nils Urbach

Over the past decades, digital technologies have changed the ways companies are doing business. In this context, particularly software’s importance for the corporate world has grown substantially (Andreessen 2011). Already a few years ago, Scott Farquhar, Atlassian’s co-founder, emphasized that “companies now only fit into two buckets: either becoming a software company, or being disrupted by one” (White 2014). Although such a statement may be biased, seen with the eyes of a corporate software vendor, he was mostly right in his assessment of the changing power structures of the corporate world. Today, software-based (digital) innovations are both crucial and a key market differentiator for many businesses (Legner et al. 2017). The value creation processes of many successful companies center around and depend on software. At the same time, the application fields of software in the corporate world have changed. While in the past software was mostly used to support business processes, its roles have shifted to become a company’s product (for software companies such as SAP or Microsoft), an inherent part of product-service bundles (for digital service providers such as SONOS or Tesla), or the basis for entire business models (for digital businesses such as Google or Facebook). Nowadays, we see that particularly companies that place software at the center of their business attain success; they regard software development as their core competency and everything else as a replaceable commodity. In this sense, Tesla considers itself a software company and a leader in utilizing artificial intelligence (AI) for autonomous driving rather than a car manufacturer (Aziza 2019).

Software’s increased and increasing importance in business has several implications for corporate and IT managers, both at the strategic and operational levels. Concerning the strategic implications, we can consider software to be a dominant driver of *servitization*, the transformation process that shifts a company’s business model and logic from product-centric to service-centric (Kowalkowski et al. 2017). In this context, digitalization enables firms to reconfigure their service business, in that services that previously required local presence and high customer interactions can increasingly use back-end units; further, new services are becoming increasingly software-centric (Sklyar et al. 2019). This is also why we see a considerable blurredness of boundaries between business and IT departments and an increasing fusion of the two. Already, more and more companies are following the concept of business-managed IT—the autonomous procurement, deployment, and management of IT instances by business entities in alignment with the IT department, or a split responsibility model (Klotz et al. 2019). This development is meaningful because digital innovations can only occur in close cooperation between business and IT and should ideally be created where they will be used later on—i.e., in digital business departments (Urbach and Ahlemann 2019). A strategic challenge regarding software relates to its being both a commodity and a mission-critical resource for most companies. Besides finding out in which application fields software is either a commodity or a differentiator, companies need flexibility in managing software, considering its importance in relation to among other IT infrastructures, governance mechanisms, and organizational designs, which ensure such necessary flexibility.

At the operational level, several developments are addressing the changing requirements of software-defined businesses. In software development, we see concepts such as DevOps, as the integration of development and operations to deliver software applications faster and at a higher quality than traditional approaches (Ebert et al. 2016). Further, low-code and no-code development platforms allow (non)programmers to create software applications via graphical user interfaces and configuration instead of doing so via traditional programming (Totterdale 2018). By allowing business employees without a sound software development background to build and test software, they contribute to the blurred boundaries between business and IT. To cope with the related organizational challenges and to address the quest for more agility and speed in generating (software) innovation, companies increasingly rely on agile organizational setups. Here, they can benefit from their experiences with agile IT setups (Jöhnk et al. 2017) and can transfer insights to the whole organization. Finally, many companies have realized that running a software-defined business requires skills and competencies that are not yet available in-house. In such a case, strategic partnerships with technology companies can be an option to address this shortage (Urbach and Ahlemann 2019). It also seems increasingly reasonable for incumbents to evaluate and lever the potential of external innovation sources originating from startups. For instance, from the perspective of banks, Fintechs are increasingly seen not only as a source of disruption but also as an opportunity for collaboration and increased innovation (Drasch et al. 2018).

Considering the future of corporate software usage, several developments will further increase software’s importance in business environments. Software remains and will also in future be the major driver of the increasing automation of business processes. One example is robotic process automation (RPA), an approach to automating processes within a broad pool of different technologies. RPA development environments provide intuitive user interfaces that foster usability and rapid implementation of software robots. These software bots access systems and perform tasks similar to humans or imitate them, automating processes originally performed by human work (Hofmann et al. 2020). A key novelty of RPA is that software increasingly uses other software. Further, while “software is eating the world,” new technological developments are waiting in the wings to change the roles and applications of software, in the same way as cloud computing and container technologies (docker) have done in the past few years. With the further development of emerging technologies, the software industry itself could also be at risk of being eaten. In this vein, AI is considered the next disruption to “eating software” (van Attekum et al. 2019). Individual and corporate software design and development capabilities become even more important through these progressions.

The advancements in corporate software utilization also lead to new challenges. To date, software usability engineering’s key goal has been to design human–computer interfaces with high usability and user-friendliness; in this context, the roles of IT artifacts and the users are clearly defined. In the future, software will be more than a simple “IT artifact in use” and will increasingly develop into an interaction partner for human users (such as a personal assistant). This development may require a change in software design, increasingly focusing on holistic user-IT artifact interaction instead of a narrow usability perspective (Alan et al. 2020). Further, software’s growing relevance in business and the increasing need for software-based innovation for a short time-to-market will lead to even more heterogeneous IT landscapes than we find in many companies today. This development may result in some kind of *legacy problem 2.0*. To overcome this challenge, a stable IT backbone with a flexible and modular architecture that enables the rapid and easy implementation of new modules is required (Urbach and Ahlemann 2019). At the same time, we expect that a new wave of IT architectural consolidation and standardization will become necessary in order to cope with the increasing complexity.

What are the implications of software’s growing business relevance for the business and IS disciplines? As we increasingly see business and IT tasks blend in many companies, corporate management is developing into digital management. Today’s corporate challenges require solid IT know-how; further, many business administration (research) questions can simply no longer be answered without at least some IT knowledge. Thus, in teaching, basic IT skills such as programming and system design will become more and more important, also for currently non-technical programs. Moreover, a closer integration of business and IT in research is inevitable. Thus, not only will the different research domains have to interact more intensively, but research and practice will also have to collaborate more intimately so as to address the “grand challenges” of the digital age. In this context, the BISE community with its transdisciplinary and practice-oriented research focus (Buhl et al. 2012) seems well positioned at the interface between business and computer science. Yet, as a community, we must also continually defend our roles and maintain our identity between these neighboring disciplines.

## The Strategic Redesign of IT in a Digitized World

Nico Wunderlich

The increasing interlocking of business and IT in the business world questions the established structures by which IT is organized in companies. Whereas the tangible side of IT, IT infrastructure and hardware, moves into the strategic background due to stable worldwide connectivity and cost-efficient IT outsourcing services, the intangible side of managing IT and its organizational implementation is challenged by an acceleration of technical innovation cycles which result in changing market conditions. The intangible component of IT rises from a supporting factor of business to become a dynamic organizational capability of the highest order. The organization as a whole is challenged and, in particular, the strategic concepts that determine the role of IT in organizations must be examined to determine whether they can withstand and respond to the demands arising from the technological development of IT infrastructures, the amounts of (big) data, and the accompanying valuable business options. Along the lines of digital-born start-up companies, organizations with a long grown analog history have to put their entire strategic armament to the test to gain value from data and IT.

From the angle of a digital business strategy, we are discussing a shift in strategic influence over IT and changing leadership roles for IT in organizations. Since IT is growing into a key area of decision-making, those who were once purely business executives, such as CEOs, might turn into figure heads for signaling the relevance of IT top-down for a data-driven company, digital transformation, or (inter)acting in a digital business ecosystem. When IT graduates to an integral part of entire business models and economic value generation, the complementing organizational capabilities need to be revised, since those dynamic capabilities which enact a digital business strategy affect all organizational levels. Breaking this down to the operational business level, we need to consider options for the development of the IT competence of business employees and its potential for creating dynamic capabilities as a bottom-up, employee-based process. We especially should highlight cross-functional cooperation and knowledge integration as preliminary coordination mechanisms to develop these dynamic IT capabilities that are substantial for achieving sustainable success in fast-moving digital markets.

### From IT Alignment to a Digital Business Strategy

The last 25 years have been characterized by bridging the gap between business and IT. IT entered an analog world and was integrated bit by bit, more sideways as technical support rather than being introduced bottom-up by the business or prioritized top-down by the management. Even a discussion took place if investments in IT would ever be able to deliver business value or should in principle merely be utilized for basic cost efficiency, e.g., for automation. This supportive function of IT was reflected from a strategic perspective in the concept of strategic business-IT alignment, which measures the congruence of a separate IT strategy with the overall business strategy of an organization. Higher degrees of IT alignment could be reached through social alignment of the two responsible executives of the separate strategies, manifested in social interaction between CIOs and CEOs (or the remaining business managers). The organizational implementation of this business-IT relationship on managerial level had to be embodied in official formats, such as regular meetings, to keep both sides in constant contact, share a common language regarding IT, and generate profound IT knowledge of the business managers. The bridging was necessary since the executives had different professional backgrounds and competencies and still had to share a common basis of communicating and decision making for the good of the firm. This process of aligning handles IT as a sidearm or aberration, a supportive function—in contrast to the requirements in accelerated digital markets that challenge companies to promote IT as the core of dynamic organizational capabilities and underlying employee-based IT competencies.

### A Business Strategy to Compete in a Digitized World

A digital business strategy constitutes a fusion of business and IT strategy in one single concept and is particularly distinctive in enclosing digital resources to create business value (Bharadwaj et al. 2013). This fusion manifests the rise of IT from the functional level to the firm level and demonstrates the top-down relevance of IT for business. The concept of the digital business strategy addresses implementations for dynamic capabilities in several areas: the cross-functional concept breaks up functional silos and encompasses the entire organization. Firms navigate in extended digital business ecosystems, utilize network and platform effects, as well as leveraging multi-sided business models, alliances, and complementary partnerships. A scalable, agile IT infrastructure enables digital supply chains, accelerated sense and response cycles, and increased frequency of product launches. Finally, data, knowledge abundance, and accelerated and automated decision-making are instrumentalized for value generation. Through enacting a digital business strategy, firms stimulate the IT-enabled and broadly discussed dynamic capabilities, such as agility—be it organizational or information-technical—platform capabilities, digitally supported decision-making, or organizational innovativeness. While many companies struggle with “how to execute digital transformation”, research provides an answer at hand with regard to scope, scalability, (market) speed, and sources of value creation. Equipped with this toolbox, a digital business strategy provides a firm-wide guideline on what to consider for digital transformation, and how to become a data-driven company, or how to interact in digital business ecosystems.

### Who Actually Leads IT in a Digitized Company

The two concepts business-IT alignment and digital business strategy do not only differ in their structure as either coordinating two separate strategies or indorsing one fused overall strategy, they also vary as to which organizational leaders are found to be influential in their formulation. A test of both constructs within one nomological net reveals how top executives from business and IT influence both concepts differently in knowledge-intensive companies (Wunderlich 2018). For IT alignment, the results are in line with the theoretical structure of the concept, since both leaders (CIO and top business executives) influence the formation of IT alignment in comparable intensity. After a decade to overcome the gap between business and IT via IT alignment, the well-balanced influence of both the IT and the business side on business-IT alignment highlights this concept as being approved. This is in contrast to the digital business strategy: The business strategy that dynamically incorporates IT-related resources is exclusively influenced by business executives—the CIO executes zero influence in building the digital business strategy, this being confirmed by validated statistical procedures. The findings underline that the increasing relevance of IT for the entire business moves the strategic responsibility for IT from the CIO to the business executives, in particular the CEO. The highest-ranked business executive is therefore in charge of realizing the importance of IT for the company and of incorporating this paradigm into a digital business strategy in order to issue and establish a top-down guideline for the digitalization of the company. Still, an indirect influence of the CIO on the digital business strategy remains via the process of social alignment in the regarding organizations, though this communicative coordination between business executives’ and the CIO’s understanding of IT cannot disguise the CIO’s loss of strategic influence. The “digitization” of the business strategy leads to the disempowerment of the CIO in strategic questions. When establishing a digital business strategy, probably a CEO can be considered to be the highest IT leader in an organization. This change of baton for IT from CIO to CEO underlines how deeply the rise of IT as the core of business processes, business models, and entire companies effects the organizational structures around IT.

### Developing IT Knowledge on a Functional Level

The shift of influence over IT strategy from the CIO towards the CEO raises the question of the future role of a CIO. The rise of IT to strategic top-level signals the pivotal rank of IT for the entire organization—and indicates the relevance of IT competencies of business employees on the functional level. Phenomena such as the consumerization of IT describe how the business employees’ handling of IT improves, sometimes claimed as pro-workers following the appearance of prosumers as IT-competent customers in e-business. Addressing the dynamic requirements demanded by a digital business strategy to compete in digital markets, research shows that CIO practices enhance organizational innovativeness, which is one dynamic capability (Wunderlich and Beck 2018). The study especially investigates IT-enabled innovativeness, facilitated by the IT knowledge of the business-side. The CIO demonstrates a positive influence on the IT knowledge on both the managerial level as well as the functional level. However, the results exhibit that the development of business employees’ IT knowledge is especially important to achieve dynamic capabilities in highly digitized firms: Companies guided by a digital business strategy draw organizational innovativeness to a higher extent from the functional level than from the managerial level. While it had already been found that technology-oriented strategies stimulate organizational innovativeness and consequently firm performance, the study explains in particular how the dynamic capabilities of innovativeness result from the integration of business employees and their respective IT knowledge in the context of digitization. In practice, CIOs in highly digitized businesses are well advised to concentrate on reinforcing this beneficial employee-based resource of IT knowledge, underlining the CIO’s function as an essential organizational institution for mediating external information into internal knowledge and structures (Wunderlich and Beck 2017). If the CIO enhances the IT knowledge of business employees, this opens an additional role for the CIO in developing crucial functional IT competencies. Before, educating IT knowledge on the business side was found to be a relevant CIO role on the managerial level in the context of business-IT alignment only. The influence of the CIO on knowledge exchange between the business side and IT side on the functional level has so far been under-researched, but seems to be considered for further investigation into how to generate dynamic capabilities. Organizational phenomena such as forms of cross-disciplinary co-working and cultural aspects such as a positive organizational climate for IT promise positive effects, since these phenomena enable business and IT staff to cooperate in reliable organizational configurations for intensive knowledge exchange and creation. Different forms of organizational entities are conceivable and partially implemented in practice: the options commence with cross-functional IT-related project teams to work on a temporally limited task, continue with distinct organizational units such as digital innovation labs and departments named ‘digital transformation’, and end with predominantly externally organized digital incubators and accelerators. The question arises, how tightly or loosely and—taking agile and temporal aspects into account—how impermanently or continuously the cooperation manifests itself. These organizational entities bring together business employees and IT experts to interconnect their respective competencies from both sides more closely and substantially to create dynamic capabilities.

### Reconfiguring Dynamic IT Capabilities

Implementing a renewed business strategy always demands a corresponding set of organizational capabilities that allow executing what the business strategy defines as guidance. The establishment of a digital business strategy especially demands the redefinition of IT capabilities, traditionally derived from the IT department as a threefold of (flexible) IT infrastructure, technical IT skills of the IT staff, and an intangible component of managing the IT-business relationship. Against the backbone of increasingly interchangeable IT infrastructure through cost-cutting IT outsourcing processes, the relationship component of intertwining business and IT will massively expand and develop into one future source of creating sustainable competitive advantage. This intangible competence should constantly be nurtured by new information on market and technology developments. IT capabilities should reflect those dynamic aspects that transact a digital business strategy in highly competitive digital markets: In digitized companies, IT capabilities emerge additionally outside the boundaries of the IT function and should particularly involve cross-disciplinary mechanisms of knowledge exchange and integration embracing the entire organization and its business environment. In particular, the extant view of IT capabilities as acquired mainly from the IT department appears to be outdated, when crucial components for contributing to the catalog of dynamic capabilities in a digital business environment derive from the IT competence of business employees (pro-workers), cross-functional organizational configurations, and IT decisions motivated by traditionally non-IT-related entities of business.

## Footnotes

1 Abbreviations for Google, Amazon, Facebook, Apple, Microsoft and Baidu, Alibaba, Tencent, Xiaomi.
